# Cultural significance of locusts, grasshoppers, and crickets in sub-Saharan Africa

**DOI:** 10.1186/s13002-022-00524-w

**Published:** 2022-03-26

**Authors:** Arnold van Huis

**Affiliations:** Laboratory of Entomology, Wageningen University and Research, P.O. Box 16, 6700 AA Wageningen, The Netherlands

**Keywords:** Art, Cricket, Ethno-entomology, Ethno-medicine, Grasshopper, Insects as food, Locust, Religion, Superstition, Toys, Literature, Proverbs

## Abstract

**Background:**

In sub-Saharan Africa, there is a wealth of information about insects which is often only orally available. The purpose of the study was to remedy this shortcoming and make an overview of how orthopteran species are utilised, perceived and experienced in daily life across sub-Saharan Africa.

**Method:**

Ethno-entomological information on Orthoptera in sub-Saharan Africa was collected by (1) interviews with more than 300 people from about 120 ethnic groups in 27 countries in the region; (2) library studies in Africa, London, Paris and Leiden; and (3) using web search engines.

**Results:**

More than 126 species of crickets, grasshoppers, and locusts have been identified as edible in sub-Saharan Africa. Some toxic species, such as *Zonocerus* spp., are eaten by some groups who use processing and detoxifying techniques. The katydid *Ruspolia differens* is very popular as food in central and eastern Africa and is captured by indigenous and commercial methods. Vernacular names refer to their morphology, behaviour, characteristics or the beliefs associated with the insect. The aposematic pyrgomorphid species, such as *Zonocerus* spp., are often used as medicine. Children play with grasshoppers, by for instance herding them like cattle, and they consider cricket-hunting for food as a game. The doctrine of signatures probably plays a role, as crickets, because of their chirping, are used to improve the sound of a music instrument, or as medicine to treat earache. Locust plagues are considered a punishment which requires repentance, but also an opportunity to acquire food. Proverbs and stories relate to using the orthopterans as food or to the underground lives of the crickets. Possible explanations are given as to why so many practices, beliefs and stories about orthopterans are so widespread in sub-Saharan Africa. The relevance of recording such ethno-entomological practices is discussed.

**Conclusion:**

Grasshoppers, locusts and crickets, although they may be agricultural pests, are very popular as food. They are also used in medicine, and as toys, and they play a role in religion, art and literature.

## Introduction

**Fig. 1:**
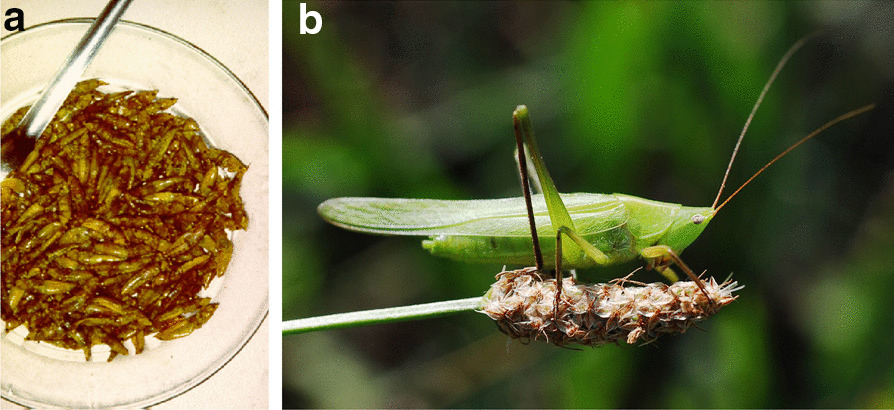
*Zonocerus variegatus*. (Attribution: Charles J. Sharp, CC BY-SA 4.0 <https://creativecommons.org/licenses/by-sa/4.0>, via Wikimedia Commons)

The commonly known terrestrial insects commonly known as short-horned grasshoppers, katydids, bush crickets, crickets, and locusts belong to the order of the Orthoptera. The order can be identified by the characteristic hind legs, developed for jumping. The metamorphosis is gradual or hemimetabolous, and the life cycle consists of eggs, nymphs, and adults. The majority are phytophagous. The number of species in the order and families below is mentioned between parentheses using GBIF [29]. The orthoptera (29,267) comprise two suborders: the Ensifera (crickets and long-horned grasshoppers) and the Caelifera (short-horned grasshoppers and locusts). The most important families from the Ensifera mentioned in this article are: Gryllidae (true crickets) (3288), Gryllotalpidae (mole crickets) (126); Stenopelmatidae (sand crickets) (57); and Tettigoniidae (katydids, bush crickets or long-horned grasshoppers) (7873). One of the most important tettigonids in this article is the edible *Ruspolia differens*, which occurs in central and east Africa. The most important families from the Caelifera are: (1) Acrididae (6831) (short-horned grasshoppers), Anostostomatidae (king crickets) (282); Pamphagidae (toad or stone grasshoppers) (418); and Pyrgomorphidae (gaudy grasshoppers) (491). Among the Acrididae are the locusts, who differ from grasshoppers; in that they can change their behaviour. Locusts are normally solitarious (like grasshoppers), but in crowded conditions, they become gregarious (different morphology, colour, and behaviour). When gregarious, the larvae (without wings) march in hopper bands and the adults (with wings) swarm (often hundreds of kilometres a day). In Africa, there are four locust species: the migratory locust *Locusta migratoria* (Linnaeus, 1758), the desert locust *Schistocerca gregaria*, (Forskål, 1775), the red locust *Nomadacris septemfasciata* (Serville, 1838) in Eastern Africa and the brown locust *Locustana pardalina* (Walker, 1870) in southern Africa. Some species of the Pyrgomorphidae show striking aposematic colouration and may eject toxic substances to warn off predators [74], such as *Zonocerus* spp., e.g. *Z. elegans* (Thunberg, 1815) (Fig. 1) sequesters pyrrolizidine alkaloids from Leguminosae and *Z. variegatus* (Linnaeus, 1758), cannabinoids from Cannibinaceae [8].

The article has a materials and methods section describing how the interviews in sub-Saharan Africa were conducted and how literature was collected. In the next section, results and discussion are combined because when presenting the results of the interviews, the information is complemented and compared with the results of the literature search. This is to give a more complete overview of the different topics, which are orthopterans as food, pests, medicine, toys, prey in hunting and in the prediction of events, and in religion, art, music, and literature. The first time an insect species is mentioned in the text, the orthopteran family is mentioned in parentheses. At the end, some conclusions for the whole study are drawn.

## Materials and methods

The information was collected by reviewing the literature and by personal interviews. The interviews were conducted in the years 1995 and 2000 in Africa and concentrated on the traditional, nutritional, and medical uses of arthropods and their products as well as on their role in religion, witchcraft, art, song, music, dance, children’s games, mythology, and literature. Although the information was collected some 20 years ago, the information is not only valid, but would probably also be more difficult to obtain now. Rapid urbanisation and the fact that older people often had to be consulted by the interviewees are signs that this type of indigenous knowledge is rapidly disappearing.

Some of the results obtained in 1995 on insects has been published [88], as well as the part on edible insects over both years (1995 and 2000) [89]. Similar articles on the orders of Coleoptera, Hymenoptera, Isoptera, Lepidoptera have been published [91–95].

The total number of people interviewed was 304 from 27 different countries in sub-Saharan Africa, of whom 22 were resource persons (experts without recorded ethnic affiliation) (Table 1). The ethnic group was unknown for five other respondents. The total number of ethnic groups was 121, excluding Zanzibar and Madagascar, where the ethnicities were not recorded. Names of ethnic groups were checked, mostly in Wikipedia [99] and the Joshua project [41].

**Table 1:** Number of respondents (N) per country and ethnic group

Country	Ethnic group—N	RES^1^	N
Benin	Bariba-1, Fon-4, Goun-1, Nagot-6, Popo-1, Tori-1		14
Burundi	Hutu-2		2
Burkina Faso	Mossi-4, Fula-1		5
Cameroon	Bafia-1, Bakoko-1, Bakossie-1, Bamileke-14, Banen-1, Bani-Pahuin-1, Bassas-2, Beti-Eton-1, Beti-Ewondo-1, Bolous-1, Matha-1, Tikar-1, Wimboum-1, Yambassa-1	2	28
CAR^2^	Gbaya-1, Kari-1		2
DR Congo^3^	Mbochi-1, Teke-1		2
Chad	Arabe-1, Goulaye-2, Kanembou-1, Mbaye-2, Ngambaye-7, Sara-Kaba-1, Sara-Niellim-1, Tupuri-1, Wadai-1		17
Gambia	Jola-1, Mandinka-1		2
Guinee-Bissau	Balanta-1		1
Kenya	Kalenjin-1, Kamba-4, Kikuyu-2, Luo-4, Meru-1, Somalian- 1		13
Madagascar	–		24
Malawi	Chewa-1		1
Mali	Fula-1, Mande-Malinke-1, Mande-Mandinka-1, Sarakolé-1, Senufo-2, Songhay-3, Tuareg-1		10
Mozambique	Bitonga-1, Makua-1, Nchope-1, Shona-1, Tsonga-Rhonga-2, Tsonga- Shangana-1, Tsonga-Tswa-1		8
Namibia	Damara-1		1
Niger	Djerma-1, Hausa-9, Kanuri-1, Songhai-4	1	15
Nigeria	Ebibio-1, Ebira-1, Yoruba-15, Unknown-1		18
Rwanda	Kiga-Toro-1		1
Senegal	Bainuk-1, Diola-4, Fula-1, Halpulaar-2, Lebu-1, Serer-3, Wolof-5		17
South Africa	–	6	
Sudan	Dongolawi-1, Fula-1, Gaälien-3, Kambari-Abadi-1, Kawahla-1, Kuku-1, Mahas-1, Nubian-1, Nubian-Mahas-1, Rubatab-2, Tunyur-1, unknown-4	5	18
Tanzania	Chaga-7, Digo-1, Iraqw-3, Iramba-1, Mwarusha-2, Pare-1, Rangi-1, Sukuma-2, Zanaki-1	1	19
Togo	Akebu-1, Ewe-5, Cotocoli-1, Kabye-1, Mina-1	3	9
Uganda	Acholi-1, Banyankole-1, Bunyoro-1, Busoga-1, Ganda-7, Langi-1, Luo-2, Nyoro-1		15
Zambia	Bemba-1, Ila-1, Lovale-1, Lozi-2, Lunda-1, Namwanga-2, Nyanja- Chewa-1, Tonga-10, Tumbuka-1	2	20
Zanzibar	–		9
Zimbabwe	Ndebele-1, Shona-9, Zezuru-1	2	11
Total number of resource persons and respondents		22	282

^1^Resource persons

^2^Central African Republic

^3^Democratic Republic of Congo

Most of the people interviewed were scientists or technicians trained in entomology. The interviewees were identified by visiting entomological groups of universities and (inter)national agricultural research institutes, plant protection services, museums, and crop protection projects. The author tried to interview most of the staff of these organisations (often arranged by the managers/directors in charge). The age of those interviewed varied between 25 and 65. Most of the respondents were male, reflecting the gender composition of the organisations. On a few occasions, people with no entomological background were interviewed in villages. This proved to be a challenge because of language and confusion about the insect species. Twenty-two of the respondents acted as resource persons on special topics (for example, experts on termites or insects as food or medicine) or had special positions (professors, heads of organisations, shamans, museum directors, and priests). In these cases, the ethnic origin of the person who provided the information was not considered relevant.

The interviews were informal and face-to face; very seldom, a group interview was conducted. The interview was semi-structured using a framework of themes (such as insects as food, medicine, toy, sign, witchcraft, poison, proverbs and insect products) and a list of insect groups (e.g. locusts, mole crickets in the case of Orthoptera). The procedure was informed consent, and participation was voluntary. Interviewees were numbered to assure confidentially. Interview transcripts in this qualitative research were analysed inductive, using both thematic content analysis and narrative analysis. Several respondents were sent this framework before the country was visited. Often, they questioned elders, grandparents, family members, and acquaintances before my arrival. This information was then passed on to me. In general, respondents from rural areas were able to provide more information on the topics of interest than those from urban areas. Findings for a country or a certain tribe were only reported if information was received from more than one respondent, or if the information given during interviews was confirmed in the literature. The respondents’ countries and tribes are mentioned to specify the sources of information. They cannot be used for establishing correlations between ethnicity and information provided. The qualitative character of the information collected is emphasised. Vernacular names and their meaning were double checked with the respondents and sometimes by a literature search.

The national libraries and university libraries in London and Paris, the library of the African Studies Centre in Leiden, the Netherlands, and some libraries of the countries visited were consulted. The literature consulted was mainly of an anthropological nature. Literature was also identified using web search engines.

## Results and discussion

### Vernacular names

**Fig. 2:**
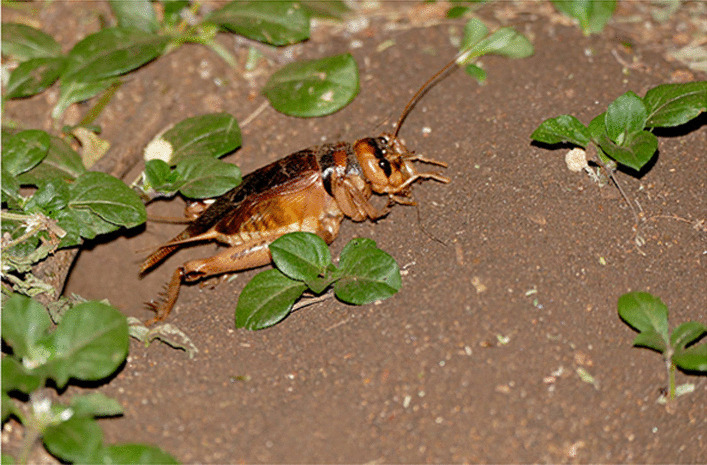
Adult of the cricket *Brachytrupes membranaceus* (Attribution: Bernard DUPONT from FRANCE, CC BY-SA 2.0 <https://creativecommons.org/licenses/by-sa/2.0>, via Wikimedia Commons)

In folk taxonomy, often common names are used which follow no formal rules with the advantage of ease of usage and common understanding among ethnic groups. For example, Ouedraogo [66] reports from Burkina Faso vernacular names of grasshoppers among the Mossi, which relate either to morphology, behaviour, characteristics or the beliefs associated with the insect. From the examples given by this author, it appears that one grasshopper may have several local names: the Mossi name of *Truxalis grandis* Klug, 1830 (Acrididae) relates to the long body and another to the protruding eyes; in the case of *Z. variegatus*, the name means that when you eat it, you will not make it till the next day, but it is also named the ‘witch cricket’ which means that you should not eat it for its poison; in the case of *Kraussaria angulifera* (Krauss, 1877) (Acrididae) the name is ‘blacksmith grasshopper’ because of its colour, but another name not only relates to its colour but also to the physogastry, as the captured females are very much appreciated when full of eggs; *Ornithacris turbida* (Walker, 1870) (Acrididae) is called ‘the master’, probably because the large insects with brightly coloured wings are difficult to catch and another name is ‘paternal orphan’ as they can only be eaten by paternal orphans. In the DR Congo (province Kwango) the very much appreciated edible insect *Brachytrupes membranaceus* (Drury, 1773) (Gryllidae) is called ‘tube of fat’ [1, p. 496], likely because of its swollen abdomen (see Fig. 2).

In Bantu languages in southern Africa, the vernacular name for *Ornithacris pictula (magnifica)* (Bolívar, 1882) (Acrididae) refers to a king grasshopper, because of its large size and the red colour of the wings (Mbunda kings in Angola had a red dress), while the large *B. membranaceus* is called the king cricket (Carl-Axel Silow: personal communication). In southern Africa, the name in Bantu languages for *Gryllus bimaculatus* De Geer, 1773 (Gryllidae) is ‘talkative wife cricket’ because of the never-ending chirping sound (to be heard at https://bit.ly/3heuo8b) (Carl-Axel Silow: personal communication).

The use of these names reflects the diversity of ways in which the natural world can be ordered and at the same time provide information of the behaviour, morphology, use (e.g. as food), and believes associated with the insect.

### Food

In the world list of edible insects [40], the number of orthopteran species as percentage of all edible species in the Afrotropical realm (24%) is higher than that in the Australasian, Nearctic, Neotropical, Oriental, and Palearctic ecozones, and higher than that in the world (13%). This shows the importance of crickets, grasshoppers and locusts as food in sub-Saharan Africa. This also is clear from historical records. Diodorus Siculus, who lived in the time of Julius Caesar, gets the credit for first describing the ‘Acridophagi’ or locust (*L. migratoria*) eaters of Ethiopia [7]. In many regions of South Africa, the same author reports that flights of locusts are looked on as such a blessing that the medicine man sometimes promises to bring them, instead of rain, with his incantations. Khoikhoi, the non-Bantu-speaking indigenous nomadic pastoralists of South Africa, enjoy eating locusts (very likely the brown locust, *L. pardalina*), especially the gravid females [27], and according to [81], they explain locust years as follows: ‘A master magician in the far north lifts a stone covering a deep trench. From the trench the locusts come out to become their food’. In southern Africa, the brown locust is considered a devastating pest, already mentioned from Botswana by Andersson et al. [3, p. 124].

In sub-Saharan Africa, in particular the Sahelian region, grasshoppers, locust, and crickets are a readily available, affordable, cheap and delicious source of protein.

### Species eaten

In Niger, when interviewing the Plant Protection Service in that country in 1995, I was told that women made more money by selling the grasshoppers from the millet than by selling their millet [90]. In the same country, it appeared through interviews that women in villages were able to name (vernacular names) many more grasshopper species than men, very likely because the women collect and prepare them as food [20]. Many grasshopper and locust species are eaten all over sub-Saharan Africa and using my interviews and existing literature I came up with a list of 97 genera and 126 species (Table 2). This number of species recorded in Africa is higher than that listed (99) by Jongema [40]. Consumption of Orthoptera varies a lot between ethnic groups. There were even differences in opinion between members of the same ethnic groups as to whether certain locust or grasshopper species are eaten. Some expressed clearly that the smelly *Zonocerus* species is not eaten. However, in the eastern part of Nigeria, in particular the states of Kwara and Ondo (towns Ikare and Owo) the eating of *Z. variegatus* is very popular (Nigeria: Yoruba), and apparently also in the centre of Cameroon [79], and *Z. elegans* for the Pedi of South Africa, precisely because of the bitter astringent flavour [73].

**Table 2:** Genera and species of edible orthopterans found by interviews (country and ethnic group) and by the literature search

Family and species	Interviews
CAELIFERA (grasshoppers, locusts)	
Acrididae	
*Acanthacris ruficornis* (Fabricius, 1787)^6,8,11,12,13^	Chad: Ngambaye; Niger: Hausa; Zambia: Tonga
*Acanthoxia gladiato* (Westwood, 1841)^8^	
*Acorypha clara* (Walker, 1870)	
*Acorypha decis* (Walker, 1870)^14^	
*Acorypha glaucopsis* (Walker, 1870)^8^	
*Acorypha nigrovariegata* (Bolívar, 1889)^8,13;a^	
*Acorypha picta* Krauss, 1877^8^	
*Acrida* sp.^13,14^	
*Acrida bicolor* (Thunberg, 1815)^8,13^	Chad: Tupuri
*Acrida madecassa* (Brancsik, 1893)^14^	
*Acrida subtilis* Burr, 1902^14^	
*Acrida sulphuripennis* (Gerstaecker, 1869)^8,13^	
*Acrida turrita* (Linnaeus, 1758)^8^	Uganda: Ganda
*Acridoderus strenuus* (Walker, 1870)^13^	Niger: Hausa
*Afroxyrrhepes procera* (Burmeister, 1838)^8,11^	
*Aiolopus thalassinus rodericensis* (Butler, 1876)^8,14^	
*Aiolopus thalassinus thalassinus* (Fabricius, 1781)^13^	
*Amblyphymus* sp.	
*Anacridium* sp.	Sudan: Kambari-Abadi, Gaälien, Kuku, Rubatab, Fula; RES
*Anacridium burri* Dirsh & Uvarov, 1953^8,13^	
*Anacridium melanorhodon* (Walker, 1870)^5,6,8,13^	Chad: Ngambaye; Mali: Sarakolé; Sudan: Kuku
*Anacridium moestum* (Serville, 1838)^8^	
*Anacridium wernerellum* (Karny, 1907)	Mali: Sarakolé
*Atractomorpha acutipennis* (Guérin-Méneville, 1844)	
*Brachycrotaphus tryxalicerus* (Fischer, 1853)^8^	
*Calephorus ornatus* (Walker, 1870)^14^	
*Cataloipus cognatus* (Walker, 1870)^8^	
*Cataloipus cymbiferus* (Krauss, 1877)^8,13^	
*Cataloipus fuscocoeruleipes* Sjöstedt, 1923^8^	Chad: Ngambaye; Senegal: Wolof
*Catantops melanostictus* Schaum, 1853^8^	
*Catantops quadratus* (Walker, 1870)^8^	
*Catantops stramineus* (Walker, 1870)^8^	Sudan (Dongolawi)
*Catantopsilus taeniolatus* (Karsch, 1893)^5^	
*Catantopsis malagassus* (Karny, 1907)^14^	
*Catantopsis sacalava* (Brancsik, 1893)^14^	
*Chirista compta* (Walker, 1870)^8,11^	
*Coryphosima stenoptera* (Schaum, 1853)^13^	
*Cyathosternum sp.*^4^	
*Cyrtacanthacris aeruginosa* (Stoll, 1813)^1,3,5,8,13^	Cameroon: Bamileke; Nigeria Yoruba; Uganda: Ganda
*Cyrtacanthacris tatarica* (Linnaeus, 1758)^8,13^	
*Diabolocatantops axillaris* (Thunberg, 1815)	Burkina Faso: Mossi; Niger: Hausa
*Duronia chloronota* (Stål, 1876)^14^	
*Eurysternacris brevipes* Chopard, 1947^13^	
*Exopropacris modica* (Karsch, 1893)^8^	
*Eyprepocnemis plorans* (Charpentier, 1825)^8^	
*Eyprepocnemis smaragdipes* Bruner, 1910^14^	
*Finotina radama* (Brancsik, 1893)^14^	
*Gastrimargus africanus* (Saussure, 1888)^6,8,11,14^	
*Gastrimargus determinatus* (Walker, 1871)^6,8,14^	
*Gelastorhinus edax* Saussure, 1899^14^	
*Gomphocerus* sp.^12^	
*Gymnobothrus madacassus* Bruner, 1910^14^	
*Gymnobothrus variabilis* Bruner, 1910	
*Harpezocatantops stylifer* (Krauss, 1877)^8,14^	
*Heteracris* sp.^8,14^	
*Heteracris coerulescens* (Stål, 1876)^8^	
*Heteracris guineensis* (Krauss, 1890)^8,11,14^	
*Heteracris nigricornis* (Saussure, 1899)^14^	
*Hieroglyphus africanus* Uvarov, 1922^12,13^	
*Hieroglyphus daganensis* Krauss, 1877^8,14^	Chad: Ngambaye, Mbaye, Tupuri; Senegal: Wolof; Sudan: Nubian-Mahas
*Homoxyrrhepes punctipennis* (Walker, 1870)^8,13^	
*Humbe tenuicornis* (Schaum, 1853)^8,14^	
*Kraussella amabile* (Krauss, 1877)^8,14^	
*Kraussaria angulifera* (Krauss, 1877)^8,13,14^	Burkina Faso: Mossi; Chad: Ngambaye, Mbayea; Gambia: Jola; Mali: Sarikolé; Niger: Hausa; Songhai; Senegal: Wolof
*Lemuracris longicornis* Dirsh, 1966^14^	
*Locusta migratoria* (Linnaeus, 1758)^4,8,9,11,12,13,14^	Chad: Ngambaye; Madagascar; Niger Songhai; CAR: Kari; Sudan: Kuku; Tanzania: Mwarusha; Uganda: Ganda; Zambia: Tonga; Zimbabwe: Shona
*Locustana pardalina* (Walker, 1870)^8,11^	Mozambique: Bitonga, Rhonga; Zimbabwe: Shona
*Mazaea granulosa* Stål, 1876^8,14^	
*Mesopsis abbreviata* (Palisot de Beauvois, 1806)^8,14^	
*Morphacris fasciata* (Thunberg, 1815)^13^	
*Nomadacris septemfasciata* (Serville, 1838)^4,8,11,13^	Kenya: Luo; Mozambique: Bitonga, Makua, Tsonga-Rhonga, Tsonga-Tswa; Niger: Hausa; Uganda: Ganda; Sudan: Kuku; Tanzania: Mwarusha; Zambia: Namwanga, Tonga; Zimbabwe: RES, Shona
*Occidentosphena uvarovi* (Rehn, 1942)^13^	
*Oedaleus carvalhoi* Bolívar, 1889^8,14^	
*Oedaleus flavus* (Linnaeus, 1758)^8,14^	
*Oedaleus nigeriensis* Uvarov, 1926^8,14^	
*Oedaleus nigrofasciatus* (De Geer, 1773)^8^	
*Oedaleus senegalensis* (Krauss, 1877)^8,13,14^	
*Oedaleus virgula* (Snellen van Vollenhoven, 1869)^14^	
*Oxya cyanoptera* Stål, 1873^5,13^	
*Oxya hyla* Serville, 1831^14^	
*Ornithacris sp.*^15^	
*Ornithacris cavroisi* (Finot, 1907)^12^	
*Ornithacris cyanea* (Stoll, 1813)^4,8^	
*Ornithacris pictula magnifica* (Bolívar, 1882)^8,13^	Zambia: Tonga
*Ornithacris turbida* (Walker, 1870)^5,6,8,12,14^	Cameroun: Bamileke; Burkina Faso: Fula; Chad: Ngambaye; Senegal: Wolof; Mali: Mande-Malinke; Niger: Hausa
*Orthacanthacris humilicrus* (Karsch, 1896)^6,8,13,14^	
*Orthochtha venosa* (Ramme, 1929)^8,14^	
*Oxycatantops congoensis* (Sjöstedt, 1929)^8,11,^	
*Oxycatantops spissus* (Walker, 1870)^8,11,13^	
*Paracinema tricolor* (Thunberg, 1815)^8,14^	
*Parapropacris notatus* (Karsch, 1891)^8,13,14^	
*Phaeocatantops decoratus* (Gerstaecker, 1869)	
*Poecilocerastis tricolor* (Bolívar, 1912)^8,14^	
*Pycnodictya flavipes* Miller, 1932^8^	
*Rhadinacris schistocercoides* (Brancsik, 1893)^14^	
*Roduniella insipida* (Karsch, 1896)^8^	
*Rubellia nigro-signata* Stål, 1875^14^	
*Schistocerca gregaria* (Forskål, 1775)^6,8,9,11,13^	Chad: Ngambaye; Kenya: Kikuyu, Luo; Togo: Cotocoli; Mali: Mande-Malinke; Niger: Songhai; Sudan: Kuku, Nubian-Mahas; CAR: Kari; Uganda: Ganda
*Sherifuria haningtoni* Uvarov, 1926^8^	
*Spathosternum pygmaeum* Karsch, 1893^12,13^	
*Stenocrobylus festivus* Karsch, 1891^13^	
*Stenohippus mundus* (Walker, 1871)^8^	
*Trilophidia cinnabarina* Brancsik, 1893^14^	
*Tristria* sp.^13^	
*Tristria conops* Karsch, 1896^8^	
*Tristria discoidalis* Bolívar, 1890^8^	
*Truxalis* sp.^12,13^	
*Truxalis burtti* Dirsh, 1950^8^	
*Truxalis johnstoni* Dirsh, 1950^8^	
*Truxaloides constrictus* (Schaum, 1853)^4,8^	
*Tylotropidius didymus* (Thunberg, 1815)^8^	
*Tylotropidius gracilipes* Brancsik, 1895^8^	
Pyrgomorphidae	
*Chrotogonus senegalensis* Krauss, 1877*s*^8^	
*Phymateus viridipes* Stål, 1873^8,13^	
*Pyrgomorpha cognata* Krauss, 1877^8^	
*Pyrgomorpha vignaudi* (Guérin-Méneville, 1847)^8^	
*Zonocerus elegans* (Thunberg, 1815)^8,13^	Sudan: Kuku; Zambia: Nyanja, Tonga
*Zonocerus variegatus* (Linnaeus, 1758)^1,3,5,8,9,12,13^	Cameroon: Bafia, Bamileke, Bassas, Bani-Pahuin, Beti-Ewondo, Tikar, Wimboum; DR Congo: Teke; Mozambique: Makua; Nigeria: Yoruba; Togo: Ewe; Zimbabwe: Shona
ENSIFERA (crickets, katydids)	
Gryllidae	
*Acheta* sp.^8,13^	
*Brachytrupes* sp. ^1,5,14^	Madagascar
*Brachytrupes membranaceus* (Drury, 1773)^3,4,5,8,9,11,12,13,15^	Benin: Fon, Nagot; Madagascar, Nigeria: Yoruba; Togo: Akebu, Ewe; Zambia: Tonga, RES; Zimbabwe: Shona
*Colossopus* sp.^14^	
*Conocephalus* sp.^13^	
*Conocephalus affinis* Redtenbacher, 1891^14^	
*Fryerius* sp.^14^	
*Gryllus* sp.^13,14^	
*Gryllus campestris* Linnaeus, 1758	Madagascar
*Gryllus bimaculatus* De Geer, 1773^8,13^	
*Modicogryllus* sp.^14^	
*Phaneroptera nana*^7,14^	
*Phaneroptera sparsa* Stål, 1857^14^	
*Pseudorhynchus* sp*.*^13^	
*Pteronemobius malgachus* (Saussure, 1877)^14^	
Gryllotalpidae	
*Gryllotalpa africana* Palisot de Beauvois, 1805^5,8,13,15^	Benin: Bariba, Fon, Tori; Chad: Ngambaye; Madagascar; Nigeria: Yoruba; CAR: Gbaya; Togo: RES; Zambia: Tonga; Zimbabwe: Shona
Tettigoniidae	
*Acanthoplus discoidalis* (Walker, 1869)^10^	Zimbabwe: Shona
*Anabrus simplex* Haldeman, 1852^8^	
*Anoedopoda erosa Karsch, 1891*^8^	
*Conocephalus* sp.^8,12^	
*Gymnoproctus sculpturatus* Schmidt, 1990^12^	
*Lanista* sp.^8^	
*Pseudorhynchus* sp.^12^	
*Pseudorhynchus lanceolatus* (Fabricius, 1775)^8^	
Ruspolia sp.^12,14^	
*Ruspolia differens* (Serville, 1838)^4,5,8,11,13,14,15;^	Burundi: Hutu; Cameroon: Bamileke, Bassas, Bani-Pahuin, Beti-Ewondo, Matha, Tikar; DR Congo: Teke; Mozambique: Bitonga, Rhonga; Nigeria: Yoruba; CAR: Gbaya, Kari; Sudan: Kuku; Tanzania: Chaga, Digo, Iraqw, Mwarusha, Sukuma, Zanaki; Togo: Ewe; Uganda: Acholi, Bunyoro, Busoga, Ganda, Langi, Luo, Nyoro; Zanzibar; Zambia: Lovale, Lunda, RES, Tonga; Zimbabwe: RES, Shona
*Ruspolia nitidula* (Scopoli, 1786)^13^	
*Plastocorypha nigrifons (Redtenbacher 1891)*	Uganda: Ganda
*Tettigonia viridissima* (Linnaeus, 1758)^9,13^	
*Zabalius* sp.^13^	
Stenopelmatidae	
*Afrogryllacris africana* (Brunner von Wattenwyl, 1888)^8^	
*Henicus whellani* Chopard, 1950^2,5^	

All names were checked with either ADW [2], GBIF [29], or Launois-Luong and LeCoq [44] and corrected when wrongly spelled by the authors of the references used

Used references: ^1^Banjo et al. [5], ^2^Chemura et al. [17]; ^3^Fasoranti and Ajiboye [25]; ^4^Gelfand [30]; ^5^Hlongwane et al. [34]; ^6^Levy-Luxereau [46]; ^7^Loko et al. [48]; ^8^Malaisse [50]; ^9^Félix [26]; ^10^Mugova et al. [62]; ^11^Nkouka [65]; ^12^Riggi et al. [75]; ^13^Séverin and Lecoq [80]; ^14^Van Itterbeeck et al. [96]; ^15^Weaving [97]

In the tropical forest areas of southern Cameroon, 87% of the people associated grasshoppers with food [64]. Several informants from Cameroon (Bamileke) reported the consumption of *Cyrtacanthacris aeruginosa* (Stoll, 1813) (Acrididae), which are collected very early in the morning by shaking the dead leaves of the banana plant. The fallen grasshoppers are then collected from the soil. Banana may not be a host plant, but the insects’ presence on dead banana leaves (brown in colour) could be a method of camouflage as the grasshoppers are also brown with a light stripe along and on top of the body.

Because grasshoppers are consumed, farmers do not use pesticides (Senegal: Wolof). Besides, it does not seem to be profitable to use pesticides to combat grasshoppers on crops like millet [38]. Women, when working in the field (Chad: Toupouri; Mali: Senoufou; Niger: Hausa) and children (Benin: Bariba, Goun; Burkina Faso: Mossi; Cameroun: Bamileke; Nigeria: Yoruba; Togo: Ewe, Kabye) collect the grasshoppers. This is often done at the end of the season (Niger: Hausa, Songhai); the gravid females are especially popular (rich in fat and females of eggs) (Cameroon: Bamileke; Mali: Malinke; Senegal: Wolof). The harvesting often takes place early in the morning when the insects are rather immobile (Niger: Haussa). The women sell them on the market (Chad: Kanembou, Ngambaye). The sale of the grasshoppers often brings more revenue than the sale of millet (Niger: Hausa). From Senegal (Wolof), the price of one kg of meat costs about 1.4 US$ and a kg of grasshoppers 1.1 US$.

**Fig. 3:**
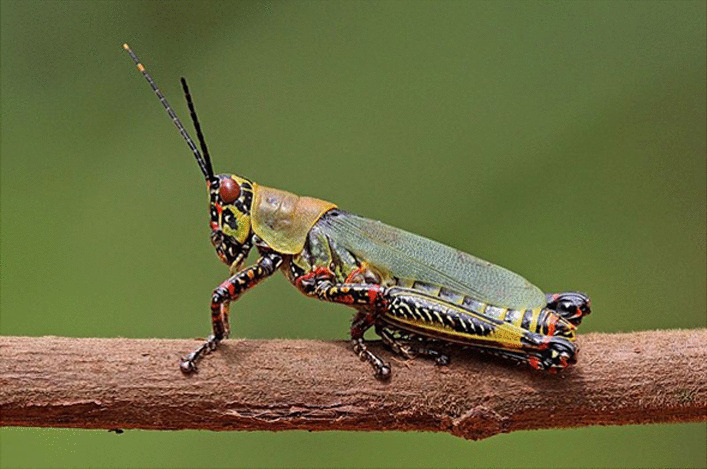
*Ruspolia differens* in Uganda. Left: meal of the grasshopper (photo by author). Right: the adult grasshopper (Attribution: Gilles San Martin from Namur, Belgium, CC BY-SA 2.0 <https://creativecommons.org/licenses/by-sa/2.0>, via Wikimedia Commons)

*Ruspolia differens* (Serville, 1838) is considered a delicacy in East Africa (Fig. 3). Especially in November, it is found in large numbers, and is caught for food. In Luganda, the language of the ethnic group Ganda, the insect is called ‘nsenene’. The insect is most prominent during the month of November, which is why this month is called 'musenene’ in Luganda [61]. The insect species is often collected by children, from November to January during the night and often from under streetlights to which they are attracted (Cameroon: Bafia, Bassas, Bamileke; Bani-Pahuin, Matha, Yambassa; RCA: Gbaya; Uganda: Ganda). Traffic accidents occur as children are so occupied with catching that they do not pay enough attention to the approaching cars (Cameroun: Bamileke; Tanzania: Mwarusha). They sometimes use butterfly nets made from shirts (Cameroun: Bafia; Tanzania: Chaga). They can also be collected from the grasslands (Cameroon: Bamileke, Bayankole, Wimbum). The recipients used are water bottles (1 1/2 L), the contents selling for about 2.2–2.7 US$ (Cameroon: Bamileke, Bassas, Yambassa); and in Uganda (Ganda) for 10 US$ per kg. The price may be higher than for meat (Uganda: Banyankole). There are also some swarms appearing in May/June but much less than the main period in November (Uganda: Ganda, Nyoro). *Ruspolia differens* is also collected commercially and en masse by using bright tube lights and corrugated iron (they glide down) (Uganda: Banyankole, Ganda, Nyoro), also mentioned by Mmari et al. [58].

The armoured bush cricket, *Acanthoplus discoidalis* (Walker, 1869) (Tettigoniidae) from southern Africa, can grow to a body length of about 5 cm of which the pronotum bears several sharp, conical spines. Their defence mechanism is reflex bleeding (also called "autohaemorrhaging") in which the insects squirt toxic haemolymph from pores in their exoskeleton. However, according to an informant from Zimbabwe (Shona) they are eaten after boiling them for 6 min, after which the water is thrown away.

Crickets such as *B. membranaceus* are collected mostly by children. This can be done very early in the morning but more often during the night when they can be localised by the sound they make (Burundi: Hutu; Cameroon: Bassas, Bolous, Wimboum; Chad: Ngambaye; Nigeria: Yoruba; Togo: Ewe; Zambia: Namwanga; Owen [67]), and often a torch is used (Cameroon: Bamileke, Bassas, Beti-Eton; DR Congo: Mbochi, Teke; Togo: Cotocoli). Full moon is the time when they are most active and best to catch (Cameroon: Bamileke, Matha; Zimbabwe: Shona). They can be collected by blocking their hole (Benin: Nagot; Cameroon: Bassas, Tikari; Nigeria: Yoruba, RES; Togo: Akebou) often with a machete (Cameroon: Bamileke) or they can be dug out the day after (Benin: Fon, Goun; Chad: Mbaye; Cameroon: Yambassa; Nigeria: Yoruba; Togo: Cotocoli; Zimbabwe: Shona). Sometimes the hole of the burrow is clogged with cotton and the crickets get entangled in the cotton and are then caught during the night (Benin: Nagot). They also introduce a grass stem and the cricket is pulled out when they bite (Cameroon: Bakoko; Mozambique: Nchope, Shona; Tanzania: Chaga; Zimbabwe: Shona). The entrance to their burrow can be found during the day from the heap of soil made by the cricket when they dig their hole (Benin: Tori; Cameroon: Banen; Togo: Ewe; Cameroon: Bakoko; [30]).

### Preparation

The legs, wings and the head of the desert locust are removed; the content of the intestines is then pushed out and the insects are barbecued, the preferred preparation among everybody in Togo (Cotocoli). When dried in the sun, they can be kept for several months. To make powder, the wings and hind legs are removed. With the meal you can make a sauce which can be eaten with a ball of cereals (Chad: Arabe). Moffat [59 pp. 448–449] mentions that after boiling, the locusts are spread on mats to dry, and are then winnowed, to clear them of their legs and wings. They are either stored in sacks as such or ground and stored as flour. Gelfand [30] describes the preparation of the migratory locust, which are collected in large baskets from 5:00 am from trees and bushes. They are poured into boiling water and then spread to dry in the sun for a day of two. Then, they are stored in the granary. Later, they are put in water and salt and stirred till all the water has gone. They are eaten as a relish with porridge. From *B. membranaceus*, they remove the wings and forelegs, but the hind legs are kept. The abdomen is opened, and the intestinal contents squeezed out and cast aside. The locusts are grilled, and salt is added. They are eaten with thick porridge.

From the variegated grasshopper *Z. variegatus* (in French ‘cricket puant’, translated as ‘stinking grasshopper’), the wings are removed as well as the last part of the abdomen to get rid of the odour; they also press the faeces out; they often boil the grasshoppers in hot water for 30 min, then salt, wash them, then fry and cook them (Nigeria: Yoruba). In Cameroon (Bani-Pahuin, Tikar), they also remove the wings and intestines, boil them for 10 min, and pass them through a colander, after which they are fried in oil. Also, Seignobos [79] mentions from the north of Cameroon the boiling in water to get rid of the odour.

Grasshoppers can be prepared in different ways. However, very often the head (with digestive tube), wings and legs are removed or when roasting the wings are burned off by the fire (Chad: Goulaye, Ngambaye; Mali: Peulh). Children often barbecue the grasshoppers in a brochette (impale them between the head and the thorax) (Mali: Malinke). They may also be fried in oil. They can also be boiled with some water until they become brown/yellow/orange (Cameroon: Beti-Ewondo; Chad Goulaye). They are then fried with salt, pepper, onion, tomato, and celery; for preserving them, they are fried a bit longer (Chad: Goulaye). They may also be put in hot water, dried, and sold for about one dollar cent per cricket (Niger: Hausa).

Cricket such as *B. membranaceus* are prepared as follows. Often the head (and with it the intestines) is removed and they can be fried without using fat when there are just a few (Cameroon: Bamileke, Yambassa; Nigeria: Yoruba; Togo: Akebu) or they are roasted (on a stick) such that the wings are burned off when there are many insects (Benin: Bariba, Fon, Goun, Nagot, Tori; Burkina Faso: Mossi; Burundi: Hutu; Cameroon: Bakossi, Banen, Matha; Tikari; Nigeria: Yoruba; Togo: Ewe). Several respondents mentioned that the femur is very delicious (Nigeria: Yoruba; Gelfand [30]) as well as the eggs (Cameroon: Bolous, Bakoko; Burundi: Hutu). In the abdomen, several cuts may be made so that they will fry well (Cameroon; Bamileke).

Mmari et al. [58] mention for *R. differens* in Tanzania many preparation methods after cleaning and washing, e.g. boiling, smoking, toasting, deep frying, sun-drying, and some preservation methods allow 12 months’ storage.

### Pests

Although the desert locust is appreciated as food, the swarms are very much feared. The collection of the locust is also a kind of plant protection (Mali: Malinke). The Nandi, part of the Kalenjin ethnic group in Kenya, plant a branch of *Lippia* sp. in the Eleusine (a cereal) fields to keep away the locusts [36, p. 86]. Charms may also be put in the fields or hung on the hedges to guard the crops against locusts [36, p. 19]. This was also mentioned in Niger (Hausa) where they put an inverted calabash in the field on a stick [see also Tremearne [85 pp. 23, 24] to which rags or bunches of leaves are tied. Muslims do the same, but they substitute leaves with sheets of paper with a prayer of the Prophet Mohammad [85], pp. 462, 463].

Cassava should always be grown, as explained by the grandmother of a respondent in Zambia (Namwanga), because when there is a plague of locusts all grains will be eaten (maize, sorghum and millet). Cassava tubers, however, are underground, and the leaves are bitter and not liked by locusts. This is confirmed by Bouvier [11] who reported from the DR Congo (province of Lomami), that during locust invasions, such as in 1931, maize fields were devastated but cassava were left almost untouched, probably because of cyanogenic glycosides found in the leaves [56]. Other grasshopper species such as *Zonocerus* spp. can eat cassava leaves and are therefore considered a pest of this crop [64]. When insects are available in large quantities people are more inclined to consider them as food (already mentioned by [27]. This also happened in Europe with large outbreaks of cockchafers which were made into a soup until the mid-1900s [54].

### Medicine

Grasshoppers are eaten not only to provide nutrition but also to cure diseases. The reason given is that these insects eat from lots of plants and trees that contain compounds that can cure diseases (Chad: Wadday; Niger: Songhai). The same reason was given in the Sudan for the cure of many diseases: diabetes, stomach cancer, jaundice, high blood pressure (Kuku) and stomach diseases (Mahas). Specifically, to cure diabetes, the tree locust, *Anacridium* sp. (Acrididae) was mentioned (2–3 locusts should be consumed daily) (Sudan: Kawahla, Rubatab). Some evidence for these statements can be found in a publication by Cheseto et al. [18] indicating that the desert locust ingests phytosterols from a vegetative diet, and amplifies and metabolises them into derivatives with potential salutary benefits.

Species of the genus *Phymateus* (Pyrgomorphidae) are African grasshoppers of about 70-mm long. These locusts feed on highly toxic plants such as milkweed (*Asclepias* and *Gomphocarpus* species) [56] (Asclepius refers to the God of medicine in ancient Greek religion). The body and forewings of the African bush grasshopper *Phymateus viridipes* Stål, 1873 are green in colour while the hindwings are bright red and blue, presenting a striking appearance in flight. They are also called ‘stinkweed locusts’ as they expel a foul-smelling odorous foam from their thoracic joints to defend themselves against predators [71, p. 94]. The foam is toxic and likely contains the poisons found in milkwood plants. In Zimbabwe (Shona), they roast the grasshoppers, grind them, and put the powder in the child’s food, to prevent bedwetting. Another species *Phymateus saxosus* Coquerel, 1861 (local name is ‘dog grasshopper’) occurs on Pervillacaea (*Menabea venenata* Baill.*)* (Asclepiadaceae), a very toxic plant, endemic to Madagascar, of which the roots are used for medical purposes such as stomach ache, venereal disease, liver problems and childhood eczema [78]. The grasshopper, which has a repulsive odour, is dried and ground into a powder to treat caries (Madagascar). In Zambia, the ashes of *Phymateus* spp, viz. *P. viridipes and P. baccatus* Stål, 1876, are rubbed into razor-blade-made scars on the breasts of women to treat breast pain [55].

Other pyrgomorphid species, viz. *Z. elegans* and *Z. variegatus* are used to ease breast pains: ashes are rubbed into scars made in the breast (Zambia: RES). In Cameroon, *Z. elegans* is used to treat diseases such as spleen pain, burns, tuberculosis, angina, and malaria [64]. In Nigeria, this species is used for curing common eye problems such as itching and redness [24]. In Tanzania, the same insect species, but also *Atractomorpha acutipennis* (Guérin-Méneville, 1844) and *Oxycatantops spissus* (Walker, 1870) (both Acrididae), are used by local people to treat similar diseases [43].

The toad or stone grasshoppers (Pamphagidae) are roasted and given to children to eat to stop them from bedwetting. They are also used to stop cancerous growth (Zimbabwe: Zezuru).

The powder of a grilled mole cricket, *Gryllotalpa* sp. (Gryllotalpidae) is put on the tongue to treat chest pains (Zambia: RES) or in water to treat cough (Senegal: Diola). Mbate [55] reported this treatment in Zambia for babies: the powder of the roasted mole cricket was rubbed into slits made on the baby’s chest. In Nigeria (Ondo State) women mix burned mole cricket with a special soup to increase the chance of pregnancy [24].

In Nigeria, the gut content of *B. membranaceus* is rubbed on infected feet for healing [24]. This insect species in Benin is also used by the Nagot in Benin for two purposes: (1) as an oral medicine (insect mixed in palm oil) for children who have difficulty speaking; and (2) against snake and scorpion bites (as powder applied topically and as decoction orally administered, respectively) [48]. Crickets are also used in a mixture with certain herbs to make sure that people can sing with a clear voice (Niger: Hausa). To cure an earache, water with powdered grilled crickets is used as an ear drop (Senegal: Lebu). In Cameroon, crickets can be used to treat mumps (smearing a paste of crushed roasted insect on the cheek), rheumatism (consuming the insect in ashy state), cramps (eating one cricket a day). Also, eating ashes of this insect improves expression in children of 2–3 years [83]. The same author mentions that grasshoppers and locusts can combat tongue inflammation in children.

In Uganda (Ganda), *Acrida bicolor* (Thunberg, 1815) (Acrididae) is used in bedwetting treatment (Uganda: Ganda), while in Zambia, both *Acrida* spp., *A. bicolor* and *A. sulphuripennis* (Gerstaecker, 1869) are used to fight hypertension by drinking the ground roasted insect in water or soup [55].

The katydid *Phaneroptera nana* Fieber, 1853, is used to treat a fungal infection of the feet (Athlete's foot) [48].

To record traditional practices used for treating a myriad of illnesses and diseases may help in finding sources of drugs for modern medicine with immunological, analgesic, antibacterial, diuretic, anaesthetic, and antirheumatic properties [70].

### Toys

As with beetles [95], children tie ropes to the legs of the grasshoppers and let them fly (Gambia: Jola; Nigeria: Yoruba; Sudan: Gaälien; Tanzania: Mwarusha; Toto: Kabye). Catching the locusts is considered a game (Tanzania: Pare; Zambia: Tonga). Children like the kicking of the spiny hind legs of locust (Tanzania: Chaga, Iraqw).

In Sudan (Fula, Gaälien, RES), children catch a locust and remove the hind legs from the body. They can move the tibia up and down by pressing the femur. Then, the game goes as follows: one says ‘O holy man pray’; somebody else answers ‘I am not praying’; then the child orders: ‘Pray!’; and then the child presses the femur and the tibia goes up and down, simulating praying in Islam. Then, it is concluded by saying: ‘O, God is great’. The same practice is used in Tanzania (Mwarusha) to indicate that somebody is calling.

Children remove the hind legs of the locust, so they cannot fly off, then they herd them like cattle (Madagascar, Sudan: Fula, Kuku, RES; Tanzania: Chaga, Iraqw, Iramba, Mwarusha, Sukuma; Zakari; Zambia: Tonga; Zanzibar). They also make a rope from sisal or banana pseudostem and tie it around the body between the head and the thorax. The locust is considered a cow which can be pulled (Tanzania: Chaga). Children playing with grasshoppers as if they were herding cattle is also mentioned in Burkina Faso [66] and the north of Cameroon [79].

Crickets are often captured by children (see subchapter on species eaten), and it is considered a game to catch the highest number of crickets (Benin: Fon; Cameroon: Bakoko; Nigeria: Yoruba; Tanzania: Chaga).

In Madagascar, fights between males of the mole cricket *G. africana* are organised by children. The capturing is a game as is the fight [21, p. 145].

### Prey in hunting

Grasshoppers are used as bait for fishing (Cameroon [64]; Chad: Sara-Niellim; Sudan: Fula; Zanzibar; Mozambique: Nchope; Zimbabwe: Shona), such as *Z. variegatus* (DR Congo: Teke; Togo: Ewe; [46] and *Z. elegans* in Mozambique (Nchope). As such they may be sold to fishermen. The desert locust is also used for fishing, but the wings are removed, otherwise they will float. The legs are also removed because the fish find them difficult to eat (Sudan: Gaalien). *Gryllotalpa africana* Palisot de Beauvois, 1805 is also used as bait (DR Congo: Teke; Zimbabwe: Shona), and again the wings must be removed otherwise they float (Zambia: Tonga).

In the Sudan (Gaälien), a small grasshopper is used to catch birds. Its forewings are taken off and they have bright yellow and red hind wings, probably attractive to the bird. It is put on a trap, attached to a string. When the insect is taken by the bird, the string releases a pin, and a door falls closing the trap. In Sudan (RES), light traps are also used. The trap is a recipient of water, with a lamp above it. The grasshoppers are fed to poultry.

In Lomami in the DR Congo, grasshoppers—very much liked as food—may be caught using a bow and special bamboo arrows [11, p. 43]. The use of bow and arrow to hunt grasshoppers is practiced in Cameroon, or they are caught by touching them with a stick with sticky glue (from *Loranthus* seeds) at the end [79]. The same author mentions that at the end of the rainy season in the north of Cameroon, *O. turbida* is caught during the night using self-fabricated butterfly nets.

### Prediction of events

Years of locust plagues are often used as a mark in history and old people refer to other events as so many years before or after that locust plague; for example, one may say ‘he was born when the locusts came’ (Chad: Kanembou. Wadai; Mali: Songhay; Fisher [28]). The Kikuyu in Kenya count ages from the date of a circumcised batch, e.g. Kyangige is the year of locusts and this was 1927 and 1932 [16]. S.W. Koelle writes in 1854 (see Fisher [28]) from the Burno region near Lake Tchad, that Kaman locusts (author: very likely the desert Locust *S. gregaria*) have been related to years of famine, because they leave no crops in the field. However, the arrival of the Difu locust (author: very likely the tree locust *Anacridium* spp.) has been associated with no famine, no grievous epidemic, and no war. The Venda from southern Africa have 12 lunar months; the twelfth is called 'Nyendavhusiku' (a kind of locust); swarms of this locust appear in this month [82].

The presence of large numbers of the grasshopper buzzard [*Butastur rufipennis* (Sundevall, 1850)] indicates a possible outbreak of locusts that attack crops [68].

The presence of locusts may be considered as a sign of a good harvest (Benin: Goun; Mozambique: Bitonga, Ronga; Niger: Hausa; Senegal: Bainuk, Wolof; Sudan: RES; Tanzania: Chaga, Mwarusha; Zimbabwe: Shona). This was also mentioned when there were many grasshoppers (Niger: Hausa). However, the association between locusts and hunger has also been made (Benin: Tori; Niger: Hausa; Tanzania: Chaga; Uganda: Bunyoro, Ganda; Zimbabwe: Shona). The resource person from the Desert Locust control organisation in Sudan mentioned that people in the Tokar Delta also believe in the association between a good harvest and the occurrence of locusts. However, according to him it is the other way round: a lot of rain brings both locusts and a good harvest. In Sudan (Kambari-Abadi, Rubatab), when the locusts come to the sorghum before head formation, and they eat the whole plant (only midribs remain), then the yield will be exceptionally high. So, the attack of the Desert Locust is very much welcomed. There may be several explanations: less evaporating surface which is beneficial in drought conditions, more tillering, slowing vegetative growth and stimulating generative/seed development [87 p. 116] or provision of manure from the faeces of the insects [42].

The arrival of the mole cricket *G. africana* is a bad omen (Zimbabwe: Shona) and announces either that there is no food in the house (Zambia; Bemba) or more often the death of a family member or a close relative (Cameroon: Bamileke; Uganda: Bunyoro, Ganda).

In Tanzania, the occurrence of more grasshoppers of *R. differens* in a particular year indicates less rainfall and hunger [43]. The arrival of these grasshoppers is announced by the nsenene bird, which is Abdim’s stork (*Ciconia abdimii* Lichtenstein, 1823), and which tends to follow the swarms [67].

Prediction of events by insects in other parts of the world has also been mentioned by Hogue [35] and merits investigation as people have an intricate relationship with the natural world.

### Religion and superstition

**Fig. 4:**
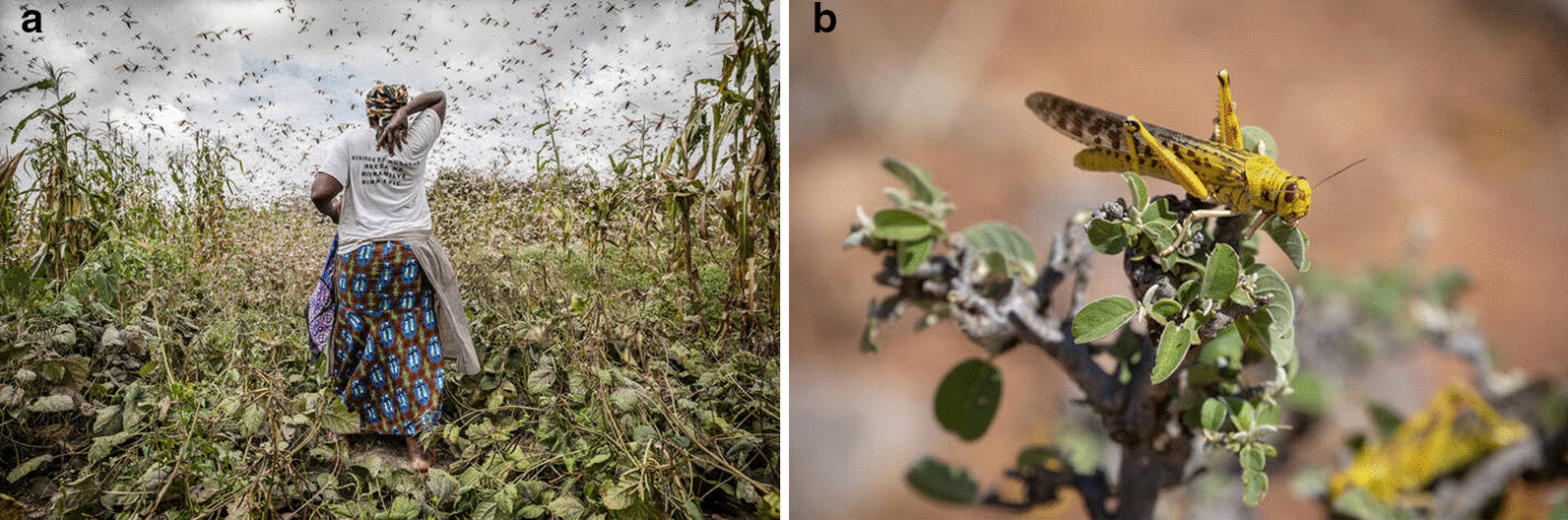
Desert locust in East Africa. Left: a maize field with a swarm. Right: an adult locust. (Attribution of photos: @FAO/Sven Torfinn)

A locust plague (Fig. 4) is seen as a calamity, but also as a punishment by God (Kenya: Kalenjin, Kamba; Luo; Senegal: Halpulaar, Serer, Wolof; Sudan: Fula; Tanzania: Iraqw; Zanzibar). To avoid hunger, people should pray, repent, conduct beer or milk ceremonies, and present meat to the poor (Mali: Songhai; Niger: Hausa, Kanuri; Nigeria: Yoruba; Senegal: Diola, Halpulaar, Peulh; Sudan: Kuku; Zambia: Tonga). Locust plagues can be brought about or ended by witch doctors (Chad: Ngambaye; Niger: Hausa), but only when receiving a certain amount of millet from the farmers (Chad: Kanembou). A number of old articles refer to locusts being sent by Gods and spirits: Tanzania (Nyamwezi) [9, p. 180], DR Congo (Tabwa) [86, p. 73], South Africa (Zulu) [12, p. 317], and Zimbabwe (Shona) [14, p. 121] and in Kenya (Kamba); and offers to spirits should be made in order to get rid of them [47, p. 509].

In Niger, the Hausa believe that *Oedaleus senegalensis* (Krauss, 1877) (Acrididae) causes pain in the ear and should therefore not be eaten; also mentioned by Levy-Luxereau [46]. *Acrida bicolor* is considered sacred (an ancestor) and should not be killed (Burkina Faso: Mossi). For the same species, it was mentioned that it is not eaten as you risk being infected with the guinea worm (Chad: Tupuri).

In southwestern Nigeria (Ogun State), the consumption of the cricket *Brachytrupes* sp. is forbidden for people from the blacksmith lineage as it caused breakage of metals during work [45]. The same reference also indicates that in Ogun male children are prohibited from eating the nocturnal crickets because it causes impotence. However, in other parts of Nigeria, it stimulates mental development and is associated with good fortune [45].

In Tanzania (Chaga, Mnyiramba, Zanaki), children are afraid of the sound of crickets as they often do not know where the noise is coming from. The cricket sound is also considered a bad omen (Tanzania: Iraqw, Sukuma) and considered dangerous as they cut crops during the night. In Zimbabwe (Ndebele, Shona) when seen in the house during the day the cricket is a bad omen. Also, in Cameroon (Wimboum, Yambassa), one should remove or kill small crickets from the house as they announce misfortune.

In Uganda (Ganda), the grasshopper ‘nsenene’ (*R. differens*) is considered a great delicacy. There are about 40 clans in the Bunyoro Kitara kingdom of Uganda, and the Abasonga, Abaikizi and Abatalaka are cattle-raising clans and have the grasshopper as their totem (Uganda: Ganda). If you belong to the same clan, you do not intermarry (you are brothers and sisters). You also do not eat the grasshopper and if you do, you may for example develop an allergy (Uganda: Ganda). The Nsenene clan has a second totem ‘Nabangogo’ which lives and feeds upon the young shoots of the plantain [77, p. 138]. I was told several stories which have to do with eating the grasshopper, with marriage and sex (Uganda: Ganda). In this regard, Roscoe [77, p. 144] mentions the following: ‘Before anyone may eat the first meal of the season, a man of the grasshopper clan must jump over his wife, or have sexual connection with her; otherwise, some members of the family fell ill’. The ceremony must take place in order that other clans may eat freely of the grasshopper, and also it would increase the number of insects. Women of the clan may prepare but are not allowed to eat the grasshopper. At the origin of the clans was Kintu, the first king of the nation. When certain animals became scarce, he ordained them taboo for certain families so they could better multiply [77], p. 137]. In Tanzania (Chaga), when you give a woman a plate of nsenene, she knows that you love her; if you give her father and mother nsenene, you can do no harm anymore; see also Mors [60]. Pregnant women in Tanzania are prohibited from eating nsenene or they would give birth to children with a coned-head like that of the grasshopper [58].

Grasshopper legs are wrapped in materials like banana leaves (fibre) and tied to the legs of a baby who is slow at starting to walk. It is believed that the infant begins walking within a short period of time, and that the children tend to walk a lot when they are grown up (Uganda: Ganda).

*Libanasidus vittatus* (Kirby, 1899) (Anostostomatidae), the Parktown prawn, is a large (6–7 cm) woodland species, which moved into the suburbs of towns (South Africa: RES). The insect gets its English name from the suburb of Parktown in Johannesburg, South Africa. As in the rainy season they are omnivorous, but they forage at night. When they arrive in the home, people go after them. They are frightening as they squirt vile-smelling faeces at their attackers [100]. It takes a long time before they are killed by insecticides. The species was elevated to a curse visiting the white man, because it only occurs in wealthy suburbs.

**Fig. 5:**
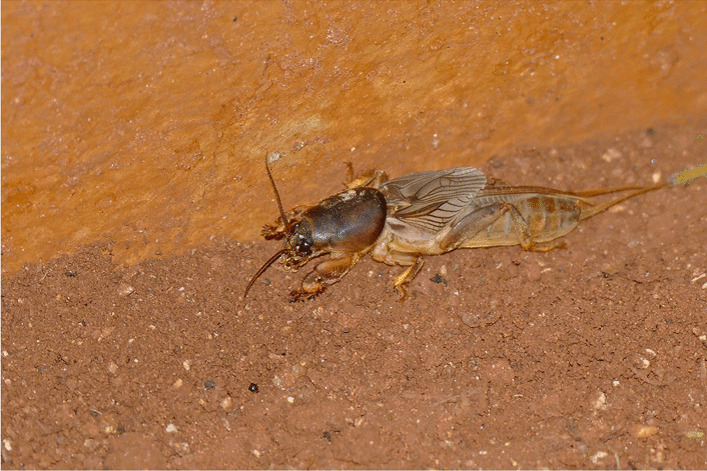
Mole cricket (*Gryllotalpa africana*). (Attribution: Bernard Dupont from France, CC BY-SA 2.0 <https://creativecommons.org/licenses/by-sa/2.0>, via Wikimedia Commons)

The mole cricket *G. africana* (Fig. 5) is removed from the house because it brings misfortune (Madagascar). However, in Zambia the mole cricket is a lucky charm and warn in a cloth around the neck, arm or carried in a pocket [55]. The Bakongo from Congo-Brazzaville, Congo-Kinshasa and Angola, hunt, cook, and eat the mole cricket [98], p. 123]. There are clans which belong to ‘heads of the mole cricket’; they are proud of their name because the mole cricket always sticks up its head even when being cooked.

The katydid *Phaneroptera nana* is used to attract a person or a customer [48]. *Zabalius* sp. and *Z. variegatus* are used for protection in Nigeria [45].

### Art and music

Ritchie [76] mentions a brass hair pin which he bought from a dealer in the town of Mopti in Mali, featuring a grasshopper which was identified as *Diabolocatantops axillaris* (Thunberg, 1815) (Acrididae). In the Sahelian region, this species can be found throughout the year in many habitats [44, pp. 47–48].

An informant from Mozambique (Nchope) mentioned that crickets are boiled in water with certain roots, after which the water is used to wash the xylophone to improve the sound of the instrument. They are also kept as amusement for the sound they make (Madagascar).

Because of their striking colouration, Pyrgomorphidae are favoured insects to be put on stamps, e.g. *P. viridipes* (Botswana, Lesotho, Rwanda, Tanzania, Zimbabwe), *P. saxosus* (Central African Republic, Madagascar), *Z. elegans* (Malawi, DR Congo, Tanzania), *Z. variegatus* (Angola, Ghana, Kenya, DR Congo, Mali) [51].

Locusts are also popular insects to be put on stamps, such as the migratory locust on a stamp of Mali commemorating the locust campaign from 1928 to 1942 (https://bit.ly/3yQiIyt). Also in Benin, a stamp was issued in 1987 depicting hand-spraying of the migratory locust [32]. In Swaziland a series of post stamps was issued in 2012, each depicting a locust species (brown, desert, red and migratory locust) (https://bit.ly/3l78Tr2).

### Literature and proverbs

Several birds have been called typical locust birds, such as the wattled starling (*Creatophora cinerea* (Meuschen, 1787)) [63, pp. 10, 13]. This has led to several proverbs used by the Kikuyu related to the bird called ‘Njũũ’ [6, p. 1, 84]:

- He is a man that looks after money as Njũũ looks after locusts (If you have a lot, you want more);
- Do not follow me as the Njũũ follow the locusts (do not follow me as you drain me as a false friend).

Other proverbs related to locust being numerous and voracious: 'Better one locust in the hand than a thousand flying' (Sudan: Dongolawi, Kambari-Abadi, Mahas, Rubatab, RES); ‘As numerous as locusts’ (Chad: Ngambaye); and ‘Are you a locust?’ means that somebody eats too much (Kenya: Kamba). A Christian book ‘Showers of grasshoppers’ by Booth [10] tells a story about fields being destroyed by locusts in Nigeria. However, the disaster was turned to their advantage when the people collected the locusts and sold them on the market as food. Pringle [72], a Scottish writer and poet, mentioned from the San in southern Africa the following song:

Yea, even the wasting locusts' swarm,

Which mighty nations dread,

To me nor terror brings nor harm -

For I make of them my bread.

In Madagascar there is a proverb ‘You cannot catch the laying grasshoppers and sleep at the same time’ [37, p. 54]. This because in the early morning it is cold and easy to catch the cold-blooded grasshoppers. A similar meaning has the proverb ‘the one who just married is not yet used to the household and goes hunting locusts in the evening’ [37, p. 150].

In Burkina Faso, the Mossi has a proverb: ‘The grasshopper holds on to the plant host, but falls when the host collapses’, meaning that when you depend on someone who loses power, you also go down [66].

In Chad (Ngambaye), it is believed the crickets come directly from the soil (they have no parents). That is why parents tell children ‘You are not a cricket’, which means you should obey us as you belong to us.

*Phymateus saxosus* is renowned as being inedible in Madagascar; it has aposematic colouration and produces an unpleasant smell when disturbed [96]. This grasshopper is called ‘dog’s grasshopper’. The grasshopper is beautiful, but it stinks and is not even liked by a dog [37, p. 198]. When somebody is a dog's grasshopper, it means that the ideas of a certain person are not appreciated and he will not be helped with his problems by his relatives or families [37, p. 166]. Concerning edible grasshoppers in Madagascar, the saying ‘The grasshoppers will not be twice near the village’ means seize the opportunity when you can [37, p. 62].

In Kenya (Masai), there is a proverb ‘Deign to catch the grasshopper for the young; when he is older he will do the same for you’ [15], p. 213] which means do good to others and good will come back to you.

In Cameroon (Beti-Eton), there is a saying ‘Nbagsana supported the bitterness of Ndole, because she accepted to eat it’ (when you start something you have to agree to finish it). Nbagsana is a dry season grasshopper and Ndole is a common name of the plant species of the genus *Vernonia* (*V. calvoana* (Hook.fil.) Hook.fil., *V. amygdalina* Delile*,* and *V. colorata* (Willd.) Drake), which are eaten as leaf vegetables. In Cameroon, the plant is a key ingredient of Ndolé, the national dish of Cameroon (https://bit.ly/3iDsAq7).

From the DR Congo, there is the following story explaining why crickets live under the ground [84, pp. 144–146]. A cricket and a lizard were friends and discussed that it was time for them to find a wife. The cricket proposed that the one who constructed the largest house should marry first and be the chief of the future village. Although the lizard objected at first, they finally agreed. The cricket made the largest house. Both got married and had many children. At a certain moment, the lizard’s family became hungry. The son of the lizard started begging the cricket for food. The cricket got angry and insulted both the son and the father of the lizard. From then on, the two were at odds. However, when the house of the cricket was inundated, the lizard invited the cricket to live in his house but told him: ‘I bear no grudge against you, but from now on you will no longer be the chief of the village’. From that moment onwards, the crickets started to live under the ground where the lizards are.

These stories provide an insight into the origin and development of human societies, cultures, and religions and according to Hogue [35] are aesthetically pleasing to study.

## Conclusions

The study through interviews and literature findings revealed that 126 orthopteran species are eaten in sub-Saharan Africa, which is a higher portion of all edible species than that on a global scale. Grasshoppers are often collected early in the morning when these cold-blooded animals are rather immobile. Harvesting is often done by women (and children) when working in the field. They are either marketed or consumed at home. Depending on the species, different preparation methods are used. For example, a preparation method of the smelly *Zonocerus* spp., which is only eaten in some areas, will get rid of the odour. Locusts, although a calamity, offer an opportunity for harvesting and consumption. Capturing crickets is more complicated and therefore often done by children, who consider it a game using several detection techniques and trapping methods. The collection of the edible tettigonid *R. differens* is very popular and is done in three different ways: from grass fields, from streetlights and commercially with light traps. The popularity of using orthopterans as food in sub-Saharan Africa and particularly in the Sahelian region has probably to do with their easy availabily, affordability, and nutritional value. The eating of insects is often considered by Western people as a primitive habit. However, it is increasingly realised in the Western world that for nutritional, health and sustainability reasons, the idea of eating insects is not bad at all. The list of orthopteran species provided may lead to find species more suitable for farming than those few currently used.

The doctrine of signatures ‘similia similibus curantur’ (likes are cured by likes) means that either the appearance or the behaviour of the insect is believed to be transferred to humans, animals or objects. For example, one of the most prominent features of the crickets is their chirping. So, cricket ingredients are used to give musical instruments a better sound. Or it is believed that crickets can cure an earache or improve speaking. The continuous chirping of one cricket species has incited people to call this insect a ‘talkative wife’. Probably, the peculiar shape of body and head of the cone-headed or long-headed grasshopper *A. bicolor* has incited people to consider it an ancestor or to use the insect as a medicine.

A large part of the world population depends on indigenous healthcare based on medicinal plants, particularly in sub-Saharan Africa [23]. In our survey and also from Zimbabwe [31], it is believed that grasshoppers that eat from many plants can cure several diseases. Also, the Pyrgomorphidae (such as *Phymateus* spp. and *Zonocerus* spp.), being very colourful (an inspiration to put them on stamps), are capable of sequestering plant secondary compounds from toxic plants [52]. The release of toxic chemicals when disturbed is probably the reason that they are used for several medicinal purposes. The doctrine of signatures may apply to the belly-shaped cricket *B. membranaceus*, used to treat bleeding in pregnancy. Also, the mole cricket is used to increase the change of pregnancy. Those traditional medicine practices may be effective as cure as it is often linked to a group’s cultural and religious values; the approach being more holistic than the reductionist way in western medicin [23]. This information should be better exploited for example in finding compounds in insects with healing properties. I did not make a comparison with practices outside sub-Saharan Africa and I refer to other publications such as those on entomotherapy [19, 57] or on nutritional value of crickets [49].

Locust plagues may have a profound effect on people’s lives, which is why years of locust plagues are used as a marker in history. As a farmer there is also little you can do about locust plagues, apart from trying to chase them away, as clearly explained in the book by Booth [10]. It is considered a punishment by God, spirits or ancestors similar to other agricultural pests such as the armyworm [92]. Another peculiar thing is that locust years are associated with good harvests, but that may be due to locust multiplication when there are abundant rains.

For children, the collection of crickets is considered a hunting game, because they are not so easy to catch. They have developed all kind of catching techniques. Grasshoppers in several countries are also used in a game in which they are treated as cattle. Or the children let them fly on a string.

Taboos on eating grasshoppers are especially the case for *R. differens* in Tanzania and Uganda. The reason for taboos is often the protection of a certain species. Some crickets, such as the giant Parktown prawn in South Africa, scare people because of their size, aposematic appearance, their jumping capacity and ejection of faecal liquids when threatened.

The stories, proverbs and songs about grasshoppers and locusts often refer to their edibility, and in the case of crickets to their living underground.

It is surprising that so many beliefs and stories about orthopterans are so widespread in sub-Saharan Africa. Why did this happen? This notwithstanding the fact that there are more than 2000 ethno-linguistic groups in Africa, of which the most important are the Niger-Congo group (1650), followed by the Afro-Asiatic (200–300), The Nilo-Saharan (80), and the Khoisan (40–70) [99]. Could there be a common origin? Bantu peoples of the Niger-Congo group occupy almost the entire southern projection of the African continent [22]. Although the Bantu people spread east and southwards from the present-day Cameroon-Nigeria border, the Encyclopaedia Britannica finds the cultural patterns of Bantu speakers extremely diverse. Hewlett et al. [33] give three explanations for cultures sharing the same ideas and practices: (1) cultural diffusion, borrowing or diffusion of the ideas and practices from neighbours; (2) local adaptations, in which individuals develop similar practices and ideas to adapt to similar natural and social environments, and (3) diffusion, the movement of peoples and their ideas and practices to new areas. All three probably play a role and it is difficult to know which one is most significant.

The information collected is about recording orally transmitted ethno-entomological information which otherwise may be lost. What are the reasons for losing this kind of information? According to Jimoh [39] and Owusu-Ansah and Mji [69] Western epistemology systematically devalues, side-lines, ignores, and suppresses African indigenous knowledge systems by presenting African intellectual enterprise as illogical and sometimes primitive. Several authors [4, 13, 23] blame colonialism and post-colonial education for destabilizing and eroding indigenous structures by imposing Western ideas of governance, economy, religion and general ways of life. Globalisation is also often mentioned as having a negative effect on cultural heritage development and preservation in Africa, in which quantitative terms (economic growth and development) are considered more important than qualitative dimensions (spiritual and cultural values) [53]. Jimoh [39] proposes an African indigenous epistemology, which means to investigate, understand, assimilate, and attribute a conception of reality that is distinctively African and philosophical. There are several other reasons for the erosion of indigenous knowledge such as breakdown of the traditional family structure, human displacements, and the impact of social media [23]. It is recommended that ethno-entomological knowledge in sub-Saharan Africa is better documented, researched and exploited than currently is the case.

## Data Availability

The data that support the findings of this study are available from the author upon reasonable 538 request. All data relating to the Hymenoptera generated or analysed during this study are included in this published article.

## Abbreviations

CAR: Central African Republic
DRC: Democratic Republic of Congo
RES: Resource 530 persons
Sp(p): Species
